# Autophagy activation and enhanced mitophagy characterize the Purkinje cells of *pcd *mice prior to neuronal death

**DOI:** 10.1186/1756-6606-2-24

**Published:** 2009-07-29

**Authors:** Lisa Chakrabarti, Jeremiah Eng, Nishi Ivanov, Gwenn A Garden, Albert R La Spada

**Affiliations:** 1 Department of Laboratory Medicine, University of Washington, Seattle, WA, USA; 2 Department of Neurology, University of Washington, Seattle, WA, USA; 3 Division of Medical Genetics, Department of Medicine, University of Washington, Seattle, WA, USA; 4 Center for Neurogenetics & Neurotherapeutics, University of Washington, Seattle, WA, USA; 5 Departments of Pediatrics and Cellular & Molecular Medicine, University of California, San Diego, USA

## Abstract

Purkinje cells are a class of specialized neurons in the cerebellum, and are among the most metabolically active of all neurons, as they receive immense synaptic stimulation, and provide the only efferent output from the cerebellum. Degeneration of Purkinje cells is a common feature of inherited ataxias in humans and mice. To understand Purkinje neuron degeneration, investigators have turned to naturally occurring Purkinje cell degeneration phenotypes in mice to identify key regulatory proteins and cellular pathways. The Purkinje cell degeneration (*pcd*) mouse is a recessive mutant characterized by complete and dramatic post-natal, cell autonomous Purkinje neuron degeneration and death. As the basis of Purkinje cell death in *pcd *is unresolved, and contradictory data has emerged for the role of autophagy in Purkinje cell degeneration, we studied the mechanism of Purkinje cell death in *pcd *mice. BAX null status did not suppress Purkinje neuron death in *pcd *mice, indicating that classic apoptosis is not responsible for Purkinje cell loss. Interestingly, LC3 Western blot analysis and GFP-LC3 immunostaining of degenerating *pcd *cerebellum revealed activation of the autophagy pathway. Ultrastructural studies confirmed increased autophagy pathway activity in Purkinje cells, and yielded evidence for mitophagy, in agreement with LC3 immunoblotting of cerebellar fractions. As p62 levels were decreased in *pcd *cerebellum, our findings suggest that *pcd *Purkinje cell neurons can execute effective autophagy. However, our results support a role for dysregulated autophagy activation in *pcd*, and suggest that increased or aberrant mitophagy contributes to the Purkinje cell degeneration in *pcd *mice.

## Introduction

In 1976, workers at the Jackson Laboratory reported the discovery of a novel, spontaneously occurring neurological phenotype that was inherited in a Mendelian fashion in mice [1]. This autosomal recessive mutant was named the "Purkinje cell degeneration" mouse, and was assigned a locus: *pcd*. Since the discovery and characterization of the original *pcd *mice, at least seven other *pcd *alleles have arisen, with all but one occurring spontaneously. Most *pcd *alleles, including 1J, 3J, and 5J, yield a severe phenotype [2]. This severe *pcd *phenotype is dramatic and stereotypical. By the time of weaning at around postnatal day 21 (P21), *pcd *mice display obvious awkwardness when ambulating. From four to six weeks of age, *pcd *mice develop pronounced gait ataxia, but thereafter the severe ataxic phenotype does not progress much further, and *pcd *mice have a normal lifespan. Although *pcd *mice begin with a normal complement of Purkinje cells and a normally developed cerebellar cytoarchitecure, a dramatic process of progressive cerebellar degeneration ensues at P15, resulting in the loss of > 99% of Purkinje cell neurons, typically over the course of no more than three weeks [1]. Histological studies of *pcd *mice also reveal photoreceptor degeneration, thalamic and olfactory bulb neuron loss, and male sterility [3-5].

Recombinant mapping by directed breeding, followed by evaluation of candidate genes from the critical region, led to the identification of *Nna1 *as the causal gene for *pcd *[6]. The Nna1 protein is a highly evolutionarily conserved protein, with orthologues in *C. elegans, Drosophila melanogaster*, and humans [7]. Of its predicted functional domains, the most conserved region of Nna1 contains a zinc carboxypeptidase domain, whose enzymatic activity has been demonstrated for a *C. elegans *version of this protein [8]. Despite the identification of multiple gene mutations that result in the *pcd *phenotype, and initial biochemical and molecular analysis of Nna proteins, the mechanistic basis of the dramatic Purkinje cell neuron death in *pcd *mice remains unknown. Chimera studies performed soon after the discovery and characterization of *pcd *mice have demonstrated that Purkinje cell degeneration in *pcd *mice is a cell autonomous process [9]. However, how neurons die in *pcd *cerebellum or retina is unclear.

Autophagy is a regulated cellular degradation process responsible for the turnover of long-lived proteins and organelles, and has been genetically characterized in yeast as an essential survival response in the face of starvation [10]. In higher organisms, however, the autophagy pathway has also emerged as a crucial process for maintenance of protein quality control and organelle function [11]. In mammals, autophagy is required for normal neural function, as conditional inactivation of autophagy pathway genes in the CNS results in neurodegeneration accompanied by the accumulation of proteinaceous material [12,13]. Numerous studies now suggest that enhanced autophagy action can be neuroprotective, while impaired autophagy function contributes to or underlies a wide range of neurodegenerative diseases [14]. For cerebellar Purkinje cells, normal autophagy pathway function does appear essential, as *Atg5 *gene deletion from Purkinje cells in mice yields Purkinje cell degeneration and death [15], but the time course for the ataxic phenotype and Purkinje cell loss extends over the course of one year - not five weeks. Similar results were observed upon *Atg7 *gene deletion from Purkinje cells in mice [16], and taken together, these studies reveal the indispensability of autophagy in the maintenance of axonal homeostasis and the prevention of axonal dystrophy and degeneration.

Of the available cerebellar degeneration mutant mice, *Lurcher *is a fairly close phenocopy of *pcd*, as *Lurcher *mice display post-developmental degeneration of Purkinje cell neurons over the course of four weeks [17]. But unlike *pcd *mice, *Lurcher *is a dominant mutation, and *Lurcher *mice also exhibit secondary degeneration of granule cell neurons and inferior olivary neurons. A role for autophagy in the cerebellar degeneration in *Lurcher *mice was suggested, when Yue et al. discovered that the *Lurcher *mutant delta2 subunit of the glutamate receptor can induce autophagy by aberrantly interacting with nPIST, which is in a complex with beclin-1 [18]. As a properly functioning apoptosis pathway is not required for Purkinje cell death in *Lurcher *mice [19,20], and autophagy has been implicated in the execution of "type II" cell death [10], this observation suggests that over-activation of autophagy in *Lurcher *may account for the cerebellar neurodegeneration in these mice. As the basis of Purkinje cell death in *pcd *has not been resolved, and seemingly contradictory data has emerged for the role of autophagy in Purkinje cell degeneration, we chose to characterize the mechanisms of Purkinje cell death in *pcd *mice. To accomplish this goal, we began by testing if a functional apoptosis pathway is required for Purkinje cell degeneration in *pcd *mice. We then evaluated the status of the autophagy pathway in degenerating Purkinje cells from *pcd *mice, and noted robust induction of the autophagy response. Association of autophagy pathway structures with mitochondria was a prominent feature of autophagy activation in *pcd *Purkinje cell neurons, implicating altered autophagy of mitochondria - a process known as "mitophagy". Our results suggest a connection between mitochondrial dysfunction, autophagy, and Purkinje cell degeneration in *pcd *mice.

## Methods

### Mice

We obtained *pcd*^*5J *^mice (DBA/2J-Agtpbp1-pcd^*5J*^/J; Jackson Laboratory, Bar Harbor, ME, USA), and crossed these mice onto the C57BL/6J strain background for eight generations. To obtain *pcd *mice on a BAX null background, *pcd*^*5J *^mice were crossed with BAX knock-out mice [21], that had been crossed onto the C57BL/6J strain background. Double heterozygous progeny were then backcrossed with *pcd*^*5J *^mice, and the resulting *pcd*^*5J *^homozygous - BAX heterozygous mice were crossed with double heterozygous mice to derive cohorts. To obtain *pcd *mice expressing GFP-tagged LC3, *pcd*^*5J *^mice were crossed with GFP-LC3 transgenic mice [22]. All mice were housed at the University of Washington transgenic animal facility. All experiments and animal care were performed in accordance with the University of Washington IACUC guidelines. Mice were genotyped by PCR analysis of genomic DNA using previously published primers and conditions [21-23].

### Western blot analysis

Protein lysates were obtained by homogenizing tissues 1:10 (w/v) in PBS. Equal amounts of homogenate and sample buffer (62.5 mM Tris-HCl, pH 6.8, 4% SDS, 200 mM dithiothreitol, 10% glycerol, 0.001% Bromophenol blue) were boiled for 10 min. Protein samples were resolved by SDS-PAGE, transferred to PVDF, and probed with primary antibody for 1 hr at RT. Immunoblots were then probed with horseradish peroxidase-coupled anti-rabbit antibodies at 1:10,000 dilution (Amersham), and developed with Enhanced ChemiLuminescence reagent (Amersham). Primary antibodies were used at the following dilutions: LC3 (1:1500; Novus Biologicals), cytochrome C (1:200; Santa Cruz); Cox IV (1:750; Cell Signaling), p62 (1:1000; American Research Products), and tubulin (1:20,000; Sigma). Band intensities were calculated using NIH Image-J freeware . All immunoblots were performed in duplicate or triplicate.

### Subcellular fractionation

Nuclear and cytoplasmic extracts were prepared using the NE-PER Nuclear and Cytoplasmic Reagent's kit (Pierce Biotechnology) and following the manufacturer's instructions. Mitochondria were isolated by using the Mitochondrial Isolation Kit (Pierce Biotechnology) and by following the manufacturer's instructions outlined for the reagent-based method. Mitochondrial pellets were lysed in a buffer containing 50 mM Tris-Cl pH 7.4, 100 mM KCl, 1.5 mM MgCl_2_, 1 mM EGTA, 50 mM HEPES, 10% (v/v) glycerol, 0.1% (v/v) Triton X-100, 14 mM 2-mercaptoethanol and a protease cocktail.

### Immunohistochemistry

Deeply anesthetized mice were perfused transcardially with 4% paraformaldehyde in 0.1 M phosphate buffer (PB), pH 7.4. The brain was removed, placed in paraformaldehyde for 4 hrs, and cryoprotected in 10% sucrose and then 30% sucrose in PB. Parasagittal frozen sections were cut at 30 μm thickness on a sliding microtome. Free-floating 40 μm brain sections were blocked with 10% goat serum, 1% BSA, and 0.3% Triton X-100 in PBS for 1 hr and then incubated with DAPI (Molecular Probes) at 1:10,000 dilution. After washing, the sections were mounted on glass slides and cover-slipped in a medium of 90% glycerol and 10% phosphate buffer with n-propyl gallate added at a final concentration of 0.5% to reduce fluorescent label bleaching. The sections were viewed, and digital scans were recorded using a Zeiss 510 multi-photon nonlinear optics confocal microscope system. Digital image *z*-stacks were created, and projections were made from them using the Zeiss AIM LSM software and the NIH Image J program.

### Electron microscopy

Mice were euthanized and transcardially perfused as previously described [24]. Semi-thin sections (1 μm thick) were stained with toluidine blue and analyzed for the quality of perfusion and fixation. Cerebellar tissue with evidence of remaining vascular erythrocytes in the semi-thin sections were considered inadequate perfusions and not included in the eventual analysis. Thin sections were mounted on grids and stained with uranyl acetate and lead citrate, and sections from at least 3 separate blocks per animal were analyzed. With a Philips TEM/CM 10 electron microscope, 50 - 60 Purkinje cell soma and axonal regions/cohort were photographed, scanned into Adobe Photoshop, and examined by blinded observers who had been trained to identify autophagic vacuoles and autolysosomes, based upon established criteria and sample images from the literature [25,26].

## Results

### BAX null status does not ameliorate the ataxic phenotype of *pcd *mice

To define pathways responsible for Purkinje cell degeneration in *pcd *mice, we tested the role of apoptosis in Purkinje cell degeneration and death. In neurons, both the intrinsic pathway and extrinsic pathway of apoptosis require BAX activation for execution of cell death. Given the crucial role of BAX in apoptotic cell death, we obtained BAX knock-out mice [21], and crossed them with *pcd*^*5J *^animals. Intercrosses of the resultant progeny yielded the expected Mendelian ratios of mice that were either *pcd*^*5J *^homozygous or heterozygous on the BAX null background. We compared double homozygous mice (i.e. BAX null - *pcd*^*5J *^homozygous) with *pcd*^*5J *^homozygous mice that were heterozygous for BAX null status, and also evaluated BAX null mice heterozygous for the *pcd *allele to control for an effect of BAX upon normal cerebellar function. Phenotypic analysis revealed no difference in the onset or severity of ataxia in *pcd*^*5J *^homozygous mice that do not express BAX, when compared with *pcd*^*5J *^homozygous mice that still express BAX (Table 1). Based upon our results, the *pcd *phenotype is not modified by BAX gene dosage.

**Table 1:** Bax null status does not prevent or delay ataxia in *pcd*^*5J *^mice.

**Genotype**	**Number**	**Ataxic gait?**	**Age at onset?**
***pcd*^***5J***^/*pcd*^***5J ***^; Bax +/Bax -**	12	Yes	Weaning

***pcd*^***5J***^/*pcd*^***5J ***^; Bax -/Bax -**	5	Yes	Weaning

***pcd*^***5J***^/+; Bax -/Bax -**	12	No	N/A

### Shrinkage and degeneration of dendrites precedes Purkinje cell loss in *pcd *mice

**Figure 1:**
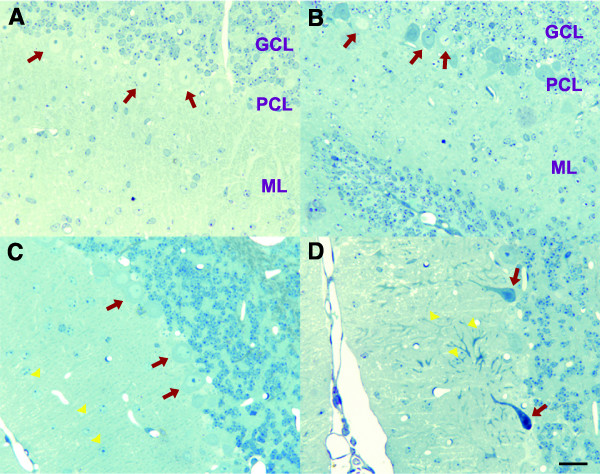
**Purkinje cell degeneration in *pcd *mice is characterized by dendrite retraction and shrinkage**. (A-D) Toluidine blue staining of cerebellar sections. At 14 days of age, Purkinje cells of normal size and morphology (red arrows) are apparent in both wild-type control littermate mice (A) and in *pcd*^*5J *^homozygous mice (B). (C) At 21 days of age, Purkinje cells (red arrows) remain regularly spaced, retain normal size and morphology, and exhibit uniformly oriented dendritic processes (yellow arrowheads) in wild-type control mice. (D) However, by 21 days of age, dramatic histopathology becomes obvious in *pcd*^*5J *^homozygous mice, as significant drop-out of Purkinje cells has begun to occur. Many of the remaining Purkinje cells have aberrantly small and misshapen soma (red arrows), and display shortened and tortuous dendrites (yellow arrowheads). GCL = granule cell layer; PCL = Purkinje cell layer; ML = molecular layer. Scale bar corresponds to 20 μM.

To better understand how Purkinje cell neurons die in *pcd *mice, we assessed the progression of histopathology in the cerebellum. As a prelude to ultrastructural analysis, we prepared semi-thin sections taken from cerebellar tissue blocks, and stained these sections with toluidine blue to verify tissue integrity and assure the success of the perfusion. We thereby confirmed that these cerebellar sections were acceptable for electron microscope analysis (data not shown). Comparison of cerebellar sections from 14 day-old wild-type littermate controls and *pcd*^*5J *^homozygous mice revealed a normal complement of Purkinje cell neurons with fine dendritic processes that extend into the molecular layer (Figure 1A-B). One week later, however, *pcd*^*5J *^homozygous mice displayed prominent cerebellar histopathology, as cerebellar sections from 21 day-old *pcd*^*5J *^homozygous mice displayed significant loss of Purkinje cells, unlike cerebellar sections from control littermates (Figure 1C-D). Of particular note, remaining Purkinje cell neurons had remarkably shortened and tortuous dendrites (Figure 1D), indicating that degeneration of the dendrites is a defining feature of the Purkinje cell pathology occurring in *pcd*^*5J *^homozygous mice. We also examined histological sections from older mice, and again confirmed that virtually all Purkinje cell neurons are lost after five weeks of age (not shown).

### Autophagy pathway activation in degenerating Purkinje cells of *pcd *mice is prominent, and is targeted to mitochondria

**Figure 2:**
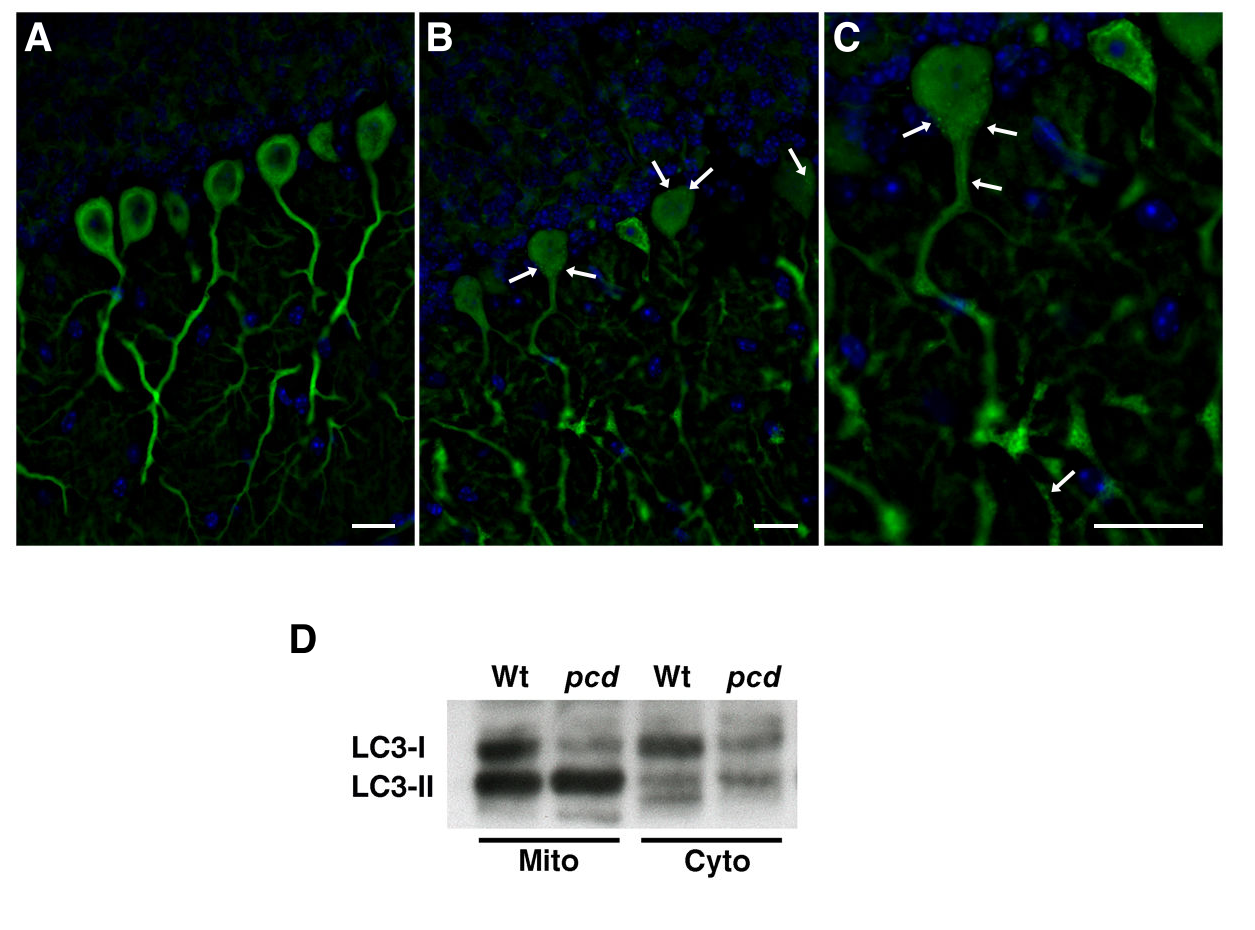
**Purkinje cell degeneration in *pcd *mice isaccompanied by activation of the autophagy pathway**. (A-C) Fluorescence microscopy of cerebellar sections from GFP-LC3 transgenic mice crossed with *pcd*^*5J *^homozygous mice. (A) At 28 days of age, wild-type mice transgenic for GFP-LC3 display a diffuse pattern of GFP staining. (B) GFP-LC3 - *pcd*^*5J *^homozygous mice, however, exhibit GFP puncta (arrows) in the cell bodies of their Purkinje cell neurons at 28 days of age. (C) These GFP-LC3 puncta are especially obvious at higher magnification, and can be seen in dendritic arbors as well as the cell body (arrows). GFP-LC3 = green; DAPI = blue. Scale bars correspond to 20 μM. (D) Western blot analysis of cerebellar protein lysates subjected to subcellular fractionation was also performed on protein samples from 28 day-old mice to assess autophagy activation. Both mitochondrial and cytosolic fractions from *pcd*^*5J *^homozygous mice showed a pronounced LC3-I to LC3-II shift. This increased LC3-II/LC3- I ratio is consistent with marked activation of the autophagy pathway in *pcd*^*5J *^homozygous mice. Note that visualized LC3 bands represent endogenous LC3 isoforms. Equivalent loading and the success of the fractionation were confirmed by re-probing the immunoblot with Cox-IV and SOD1 antibodies (not shown).

Microtubule-associated light chain 3 (LC3), the mammalian homologue of yeast Atg8, is proteolytically processed and then conjugated to phosphatidyl-ethanolamine (PE) prior to its incorporation into structures that become autophagosomes [27]. The conversion of unconjugated LC3-I to LC3-II is a useful method for assaying autophagy activation, both *in vitro *and *in vivo*, as LC3-I to LC3-II conversion can be monitored by a change in subcellular distribution, going from a diffuse to punctate appearance when in autophagosomes, or, by observing a molecular weight shift upon Western blot analysis [28]. To determine if the autophagy pathway is activated in degenerating Purkinje cells in *pcd*^*5J *^mice, we obtained transgenic mice that ubiquitously express green fluorescent protein (GFP)-tagged LC3 [22], and we crossed these mice with *pcd*^*5J *^mice. The utility of the GFP-LC3 transgenic mice for tracking autophagy activation in a wide variety of tissues has been established, as GFP-LC3 localizes to the membranes of autophagosomes [28]. When we compared *pcd*^*5J *^homozygous mice expressing the GFP-LC3 transgene with wild-type littermate controls expressing GFP-LC3, we noted prominent GFP-positive puncta in the cell bodies of Purkinje cells from 28 day-old *pcd*^*5J *^- GFP-LC3 mice, while the distribution of GFP labeling was diffuse throughout the Purkinje cells of wild-type mice expressing GFP-LC3 (Figure 2A-B). At higher magnification, GFP-LC3 puncta could be visualized in both the dendrites and cell bodies of degenerating Purkinje cells from *pcd *mice (Figure 2C).

To confirm the autophagy activation observed in histological sections and to determine if the autophagy activation process involves multiple cellular compartments, we performed LC3 Western blot analysis on cerebellar lysates from 28 day-old *pcd*^*5J *^homozygous mice and age-matched wild-type littermate controls. Immunoblotting with anti-LC3 antibody revealed a prominent LC3-I to LC3-II shift in both the cytosolic and mitochondrial fractions in the *pcd *mice (Figure 2D), suggesting that autophagosome association with mitochondria is a significant feature of autophagy activation in degenerating Purkinje cells from *pcd*^*5J *^homozygous mice. We did not observe a difference in the LC3 isoform pattern in the nuclear fraction (data not shown).

### Ultrastructural abnormalities in *pcd*^*5J *^cerebellum underscore a role for mitochondrial pathology in the Purkinje cell degeneration

**Figure 3:**
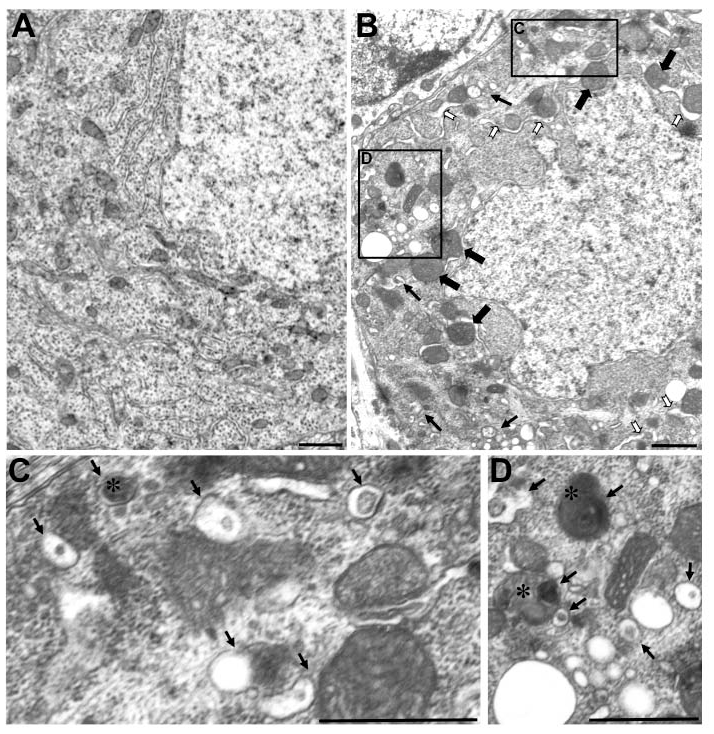
**Ultrastructural analysis of *pcd *mice reveals altered mitochondrial morphology, endoplasmic reticulum histopathology, and autophagosome engulfment of mitochondria in Purkinje cell soma**. (A-C) We performed an extensive ultrastructural comparison of Purkinje cell soma between 18 day-old wild-type and *pcd*^*5J *^homozygous mice. Representative micrographs are shown here. (A) Control micrographs from a wild-type Purkinje cell soma. (B) Purkinje cell somae in *pcd*^*5J *^homozygous develop huge mitochondria that are likely to represent fusion at early stages (filled black arrows) and very swollen endoplasmic reticulum that is largely denuded of ribosomes (white arrows). Autophagic vacuoles appear and engulf mitochondria and other organelles (line arrows). We noted a significant number of Purkinje cells from *pcd*^*5J *^homozygous mice undergoing dark cell degeneration which was accompanied by increased numbers of autophagic vacuoles. Dark cell degeneration was uncommon in wild-type control micrographs (not shown). (C) and (D) are higher magnification views of inset regions in (B). Autophagic vacuoles containing electron dense cytoplasmic elements (arrows) are present, including vacuoles containing mitochondria (asterisks). Scale bars represent 1 μM.

To more carefully explore mitochondrial histopathology in *pcd*^*5J *^homozygous mice, and to definitively characterize autophagy pathway status, we pursued ultrastructural analysis, as this method is the gold standard for autophagy analysis [27]. Cohorts of 18 day-old *pcd*^*5J *^and control mice were selected for this study, as the Purkinje cell degeneration is well underway in *pcd *cerebellum by this time point. Ultrastructural analysis yielded frequent examples of mitochondrial abnormalities in *pcd*^*5J *^homozygous mice, including enlarged mitochondria with normal electron density at early stages of degeneration, suggesting disruption in the normal dynamic between mitochondrial fission and fusion (Figure 3A-B). In Purkinje cells at the later stages of degeneration, mitochondria eventually do appear swollen with reduced electron density (Figure 3B-C). Another common *pcd *finding was abnormally dilated endoplasmic reticulum (ER) with apparent dissociation of ribosomes from the ER segments (Figure 3C-D). At later stages of degeneration, the swollen ER appears to be wrapping around mitochondria and other organelles, possibly to generate membrane that would be incorporated into autophagic vacuoles (Figure 3B-C). When we scanned electron micrographs of the cerebellum of *pcd*^*5J *^homozygous mice, we observed many Purkinje cells undergoing dark cell degeneration (Figure 3C). We also found evidence for autophagic degradation of mitochondria in Purkinje cells of the *pcd*^*5J *^mice, as we detected double-membrane bound structures that contained mitochondria (Figure 3C). These observations suggested that autophagic degradation of mitochondria, a process known as "mitophagy" [29], could be involved in the Purkinje cell demise in *pcd *mice.

**Figure 4:**
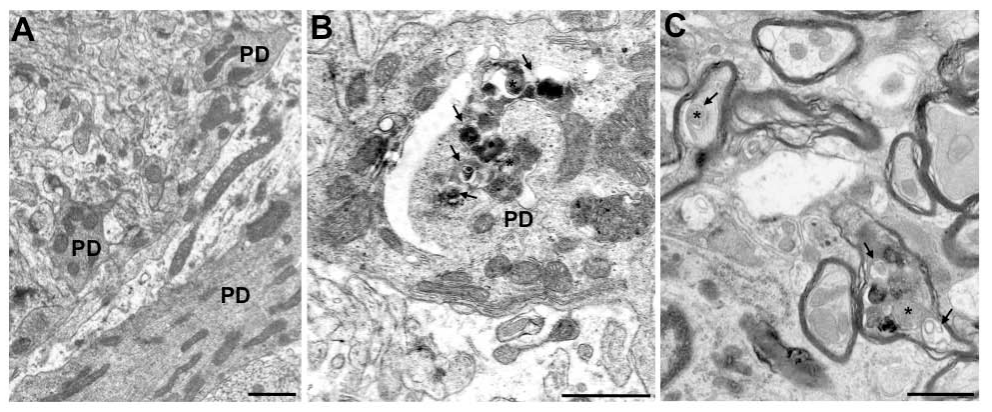
**Ultrastructural analysis of *pcd *mice indicates accumulation of autophagic vacuoles and autophagosomes in Purkinje cell dendrites and axons**. (A-B) We performed an extensive comparison of Purkinje cell dendrites between 18 day-old wild-type and *pcd*^*5J *^homozygous mice. Representative micrographs are shown here. (A) Dendrite process from a Purkinje cell neuron of a wild-type control. (B) In *pcd*^*5J *^homozygous mice, we noted frequent double-membrane bound structures in Purkinje cell dendrites (lined arrows), sometimes containing mitochondria (asterisks). (C) When we examined the Purkinje cell axons projecting to the deep cerebellar nuclei in *pcd*^*5J *^homozygous mice, we detected axons containing multiple autophagosome-like structures (arrows), containing mitochondria (asterisks) and other electron dense cellular components. Scale bars represent 1 μM.

**Figure 5:**
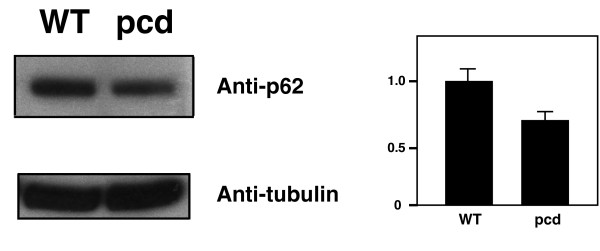
**p62 levels are decreased in *pcd *mice**. Western blot analysis of p62 in cerebellar lysates from 27 day-old wild-type littermate controls (WT) and *pcd*^*5J *^homozygous mice (pcd) revealed decreased levels of p62 in *pcd*. Densitometric ratios of p62 to the tubulin loading control are shown at right.

After surveying the cell bodies of degenerating Purkinje cells in *pcd *mice, we considered the dendrites and axons of Purkinje cells. Within these regions, we observed numerous autophagic vacuoles and autophagosome-like structures. Indeed, when we compared Purkinje cell dendrites, we typically observed multiple autophagic structures in sections from *pcd*^*5J *^homozygous mice, but noted no autophagy pathway structures in control sections (Figure 4A-B). Recent studies have shown that axonal dystrophy in Purkinje cells can result from autophagy dysfunction [15,16]. For this reason, we also evaluated Purkinje cell axons in sections of the deep cerebellar nuclei, and we noted that Purkinje cell axons of *pcd*^*5J *^mice frequently appeared to be contain autophagosomes (Figure 4C). These ultrastructural findings confirmed that the autophagy pathway is likely induced and/or blocked from completing lysosomal fusion and autolysosome degradation in degenerating Purkinje cells from *pcd*^*5J *^homozygous mice. To determine if the autophagy pathway is progressing, we chose to assess autophagy flux in the cerebellum of *pcd*^*5J *^homozygous mice. One method for monitoring autophagy flux is to measure the level of p62/SQSTM1, as p62 is associated with mature autophagic vesicles and is degraded within autolysosomes, allowing one to use p62 levels as a surrogate measure of autophagic vesicle - lysosome fusion [27]. When we compared cerebellar p62 levels between *pcd*^*5J *^homozygous mice and wild-type littermate controls, we noted a significant decrease in p62 in *pcd*^*5J *^homozygous mice (Figure 5), consistent with induction of functional autophagy in degenerating *pcd*^*5J *^cerebellum.

## Discussion

Neurons rely upon basal levels of autophagy to maintain protein quality control, and degenerate when essential autophagy pathway genes are inactivated. In neurodegenerative disorders involving protein misfolding, up-regulation of the autophagy pathway can counter proteotoxic stress and thereby retard disease progression and neuron loss [14]. For these reasons, normally functioning autophagy is believed to be neuroprotective. Although much evidence supports a role for autophagy as a protective response to misfolded protein stress in the nervous system, other data indicate that dysfunctional autophagy can contribute to neurodegeneration and neuron cell death [30]. Indeed, the association of autophagy structures and autophagic features within dying cells led to the designation of a type of cell death (type II or autophagic cell death) that became viewed as independent from the classic apoptotic cell death (or type I) pathway [10,31]. Studies of spontaneous mouse mutants of Purkinje cell degeneration have implicated the autophagy pathway in the dysfunction and death of Purkinje neurons. *Lurcher *mice, with mutations in the delta2 glutamate receptor (GRID2), display autophagy-dependent cerebellar Purkinje cell death, as mutant GRID2 can activate beclin-1/Atg6 via an altered interaction with n-PIST to induce autophagy [18,32]. However, characterization of mice with conditional inactivation of the autophagy pathway in Purkinje neurons indicates that, in the absence of autophagy, Purkinje cells will degenerate and die, likely because of impaired turnover of axonal membranes [15,16]. In this study, we examined the role of the autophagy pathway in Purkinje neuron loss in *pcd *mice. LC3 immunohistochemistry and Western blot analysis suggested induction of the autophagy pathway in *pcd *cerebellum, and ultrastructural analysis of degenerating *pcd *Purkinje cells revealed evidence for an accumulation of autophagic vacuoles in dendrites and axons. To determine if activation of autophagy in *pcd *cerebellum corresponded to a functional autophagy pathway response, we measured the levels of p62 and noted a significant reduction in p62 in *pcd *cerebellum, consistent with successful fusion of autophagosomes with lysosomes. These findings indicate that *pcd *Purkinje cell neurons are capable of carrying out effective autophagy, and that the Purkinje cell degeneration and neuron death in pcd does not stem from a failure of autophagy pathway progression.

An intriguing and unique feature of autophagy activation in the Purkinje cells of pcd mice is the association of autophagic structures with mitochondria. When we performed Western blot analysis of *pcd *cerebellar protein lysates subjected to subcellular fractionation, we noted a prominent increase in the levels of LC3-II in parallel with a marked reduction in LC3-I in the mitochondrial fraction. This shift in the LC3-I: LC3-II ratio strongly suggests that *pcd *neurodegeneration involves autophagy of mitochondria, a process known as mitophagy. Further evidence for mitophagy was present in electron micrographs of degenerating *pcd *Purkinje cell neurons, as ultrastructural analysis revealed numerous examples of mitochondria bounded by double-membrane structures. Although the regulation of mitophagy remains poorly understood, autophagic degradation of mitochondria is likely to be a selective process intended to eliminate dysfunctional mitochondria [33]. As Purkinje cells have very high energy demands, maintaining large numbers of functional mitochondria is essential for normal function and survival of Purkinje neurons. If up-regulation of autophagy in Purkinje cells in the *pcd *cerebellum is accompanied by increased levels of mitophagy, and this abnormally induced autophagy produces indiscriminate mitophagy, then Purkinje cell degeneration in *pcd *mice may result from bioenergetics collapse. Consistent with this hypothesis, we have documented decreased complex I activity in *pcd *cerebellum (Chakrabarti & La Spada, unpublished results). In Parkinson's disease, where complex I deficiency is also observed, loss of function of PTEN-induced kinase 1 (PINK1) may contribute to disease pathogenesis by promoting mitophagy, the regulation of which appears tightly linked to oxidative stress and mitochondrial dynamics [34]. Thus, Purkinje cell degeneration in *pcd *mice may not equate with a process of type II autophagic cell death, but rather may stem from dysfunctional autophagy and dysregulated mitophagy. Studies of autophagic pathology in Alzheimer's disease indicate that presenilin-1 mutations promote Aβ peptide production by impairing lysosomal function and thereby preventing autolysosome degradation of accumulating Aβ peptides [35]. As presenilin-1 is enriched on autophagosome membranes, one group has postulated that presenilin-1 could be involved in the regulation of autophagy [36]. As Nna1 possesses a GTP-binding domain and a N-terminal amino acid sequence that is predicted to promote mitochondrial localization based upon MitoProt II analysis , Nna1 could similarly be involved in regulating mitochondrial turnover through the autophagy pathway. Purkinje cell degeneration in *pcd *mice may thus reflect a cataclysmic collapse of Purkinje cell function as the energy needs of these neurons exceed ATP production capacity due to indiscriminate degradation of mitochondria, secondary to the absence of Nna1-regulated mitophagy. Although further work will determine if Nna1 is specifically involved in mitophagy regulation in Purkinje cell neurons, the findings presented herein strongly support a role for altered autophagy pathway function in *pcd*, and suggest dysregulation of mitophagy as a likely cause of the *pcd *phenotype.

## Conclusion

Degeneration of Purkinje cell neurons in *pcd *mice is characterized by activation of the macroautophagy pathway. Ultrastructural studies confirmed increased autophagy pathway activation in Purkinje cells, and Western blot analysis of subcellular fractions yielded evidence for autophagy of mitochondria (mitophagy). Purkinje neuron death in *pcd *mice likely involves over-activation of the autophagy pathway or dysfunctional autophagy, suggesting that altered autophagy pathway function could emerge as a common theme for Purkinje cell demise in a variety of neurodegenerative disease processes.

## Abbreviations

pcd: purkinje cell degeneration; GFP: green fluorescent protein; LC3: light chain 3.

## Competing interests

The authors declare that they have no competing interests.

## Authors' contributions

LC, GAG, and ARL designed the experiments. LC, JE, and NI performed the experiments. LC, GAG, and ARL analyzed the data, and wrote the paper. All authors read and approved the final manuscript.
